# Values of a novel pyroptosis-related genetic signature in predicting outcome and immune status of pancreatic ductal adenocarcinoma

**DOI:** 10.1093/gastro/goac051

**Published:** 2022-09-29

**Authors:** Xiang Xu, Jia-Hua Liang, Jian-Hui Li, Qiong-Cong Xu, Xiao-Yu Yin

**Affiliations:** Department of Pancreato-Biliary Surgery, The First Affiliated Hospital, Sun Yat-sen University, Guangzhou, Guangdong, P. R. China; Department of Pancreato-Biliary Surgery, The First Affiliated Hospital, Sun Yat-sen University, Guangzhou, Guangdong, P. R. China; Department of Pancreato-Biliary Surgery, The First Affiliated Hospital, Sun Yat-sen University, Guangzhou, Guangdong, P. R. China; Department of Pancreato-Biliary Surgery, The First Affiliated Hospital, Sun Yat-sen University, Guangzhou, Guangdong, P. R. China; Department of Pancreato-Biliary Surgery, The First Affiliated Hospital, Sun Yat-sen University, Guangzhou, Guangdong, P. R. China

**Keywords:** pancreatic ductal adenocarcinoma, pyroptosis, prognosis, immune

## Abstract

**Background:**

Pyroptosis is an emerging form of programmed cell death associated with progression in malignancies. Yet, there are few studies reporting on the association between pancreatic ductal adenocarcinoma (PDAC) and pyroptosis. Therefore, we aimed to construct a pyroptosis-related genetic signature to predict the clinical outcome and immune status in PDAC patients.

**Methods:**

RNA-seq data of 176 PDAC patients from The Cancer Genome Atlas (TCGA) and 167 PDAC patients from the Genotype-Tissue Expression Project were analysed for pyroptosis-related differentially expressed genes (DEGs) between PDAC and normal pancreas. The risk signature of DEGs was analysed using the least absolute shrinkage and selection operator (LASSO) Cox regression analysis and its accuracy was validated in the Gene Expression Omnibus (GEO) cohort (*n *=* *190). Functional enrichment analyses were performed to explore the mechanisms of the DEGs. The immune characteristics were evaluated using single-sample gene set enrichment analysis and ESTIMATE algorithms for each group.

**Results:**

A nine-gene risk signature was generated from LASSO Cox regression analysis and classified PDAC patients into either a high- or low-risk group according to the median risk score. The high-risk group had significantly shorter overall survival than the low-risk group and it was verified in the external GEO database. A nomogram based on the risk signature was constructed and showed an ideal prediction performance. Functional enrichment analyses revealed that pyroptosis might regulate the tumor immune microenvironment in PDAC. Immune infiltration evaluation suggested that immune status was more activated in the low-risk group than in the high-risk group.

**Conclusion:**

The risk signature encompassing nine pyroptosis-related genes may be a prognostic marker for PDAC. Pyroptosis might affect the prognosis of PDAC patients via regulating the tumor immune microenvironment.

## Introduction

Pancreatic ductal adenocarcinoma (PDAC), a malignancy with a dismal prognosis, accounts for the sixth commonest cause of cancer deaths in the world [1]. Due to the non-specific clinical manifestations at the early stage and lack of effective early diagnostic modalities, many PDAC patients have developed advanced-stage disease at the time of diagnosis, thus losing the opportunity for surgical treatment. On the other hand, chemotherapy for patients with advanced PDAC is frequently ineffective because PDAC cells may develop chemo-resistance through various mechanisms [2]. Hence, PDAC has a dismal prognosis with a 5-year survival rate of <9% [3].

Currently, the tumor-node-metastasis stage has been widely used for evaluating prognosis and assisting treatment decisions. However, its predictive accuracy is still unsatisfactory due to the lack of consideration of the genetic heterogeneity and subsequent biological behavior differences among PDAC patients. With the development of sequencing technology, better understanding of the transcriptome in malignancies has been achieved. Emerging evidence has revealed that molecular classifications based on genetic characteristics could have prognostic value in some malignancies including colorectal cancer [4]. Yet, due to the complexity and heterogeneity of PDAC, current understanding in molecular classifications was still far from ideal [5]. Therefore, developing new molecular classifications for PDAC prognosis is warranted.

Pyroptosis, also known as cell inflammatory necrosis, is a novel form of programmed cell death [6]. It is characterized by rupture of the cell membrane due to cellular swelling, which leads to outflow of cell content, eventually resulting in robust inflammatory response [7]. Pyroptosis is initiated by caspase-mediated cleavage of gasdermin (GSDM) family-member proteins following exogenous or endogenous stimulants [8]. In brief, the N-terminal pore-forming domain is dissociated from the C-terminal repressor domain during GSDM cleavage and then forms pores in the cell membrane, which subsequently leads to release of inflammatory factors and cell pyroptosis [9]. Recently, accumulating evidence has shown that pyroptosis may be play contradictory roles in tumors as it could be closely related to both cell death and the immune microenvironment. Pyroptosis, as a pro-inflammatory cell-death mode, might promote pancreatic cancer by maintaining an inflammation-related immunosuppressive microenvironment [10]. Meanwhile, as an innate immune mechanism, pyroptosis can inhibit the development of tumors [11]. Mammalian STE20-like kinase 1 (*MST1*) could be a potential therapeutic target for PDAC as it is under-expressed in PDAC; restoration of *MST1* expression could inhibit the cell proliferation, migration, and invasion of PDAC through caspase-1-induced pyroptosis [12]. However, scientific evidence of the association between PDAC and pyroptosis is still very limited [13].

The present study aimed to explore the expression characteristics of pyroptosis-related genes and their potential prognostic values in PDAC by generating prognostic gene signatures. In addition, we also assessed the association between pyroptosis and the tumor immune microenvironment. Our study may provide new insight for prognostic biomarkers and potential therapeutic targets for PDAC.

## Materials and methods

### Acquisition of data

The messenger RNA expression matrix of The Cancer Genome Atlas (TCGA) and Genotype-Tissue Expression Project (GTEx) were obtained from the UCSC Xena website (https://xenabrowser.net/datapages/) and the corresponding clinical features were downloaded from the TCGA database (https://portal.gdc.cancer.gov/). For further verification, the transcriptional information and clinical data were obtained from Gene Expression Omnibus (GEO) database (GSE62452, https://www.ncbi.nlm.nih.gov/geo/query/acc.cgi?acc=GSE62452, platform: GPL6244; GSE71729, https://www.ncbi.nlm.nih.gov/geo/query/acc.cgi?acc=GSE71729, platform: GPL20769). The GEO series gene-expression matrices were combined and the inter-batch difference was removed using the “*sva*” package. In addition, 57 pyroptosis-related genes (PRGs) were obtained from the REACTOME_PYROPTOSIS and GOBP_PYROPTOSIS gene sets in the MSigDB database and prior reviews [14]. All data sets used in this study are publicly available.

### Identification of differentially expressed PRGs

Due to the lack of normal pancreas tissue data in the TCGA cohort, GTEx data were also used to identify the pyroptosis-related differentially expressed genes (DEGs). The “DESeq2” package was utilized to screen DEGs with a *P*-value < 0.05. The protein–protein interaction (PPI) network of the DEGs was constructed using Search Tool for the Retrieval of Interacting Genes (STRING), v11.5 (https://cn.string-db.org/) with default parameters (confidence = 0.4). The correlations of DEGs were generated by the “corrplot” package to illustrate a correlation matrix diagram.

### PRGs-based classifications of PDAC patients in the TCGA cohort

In order to explore the molecular classification of TCGA PDAC cohorts based on the expression of pyroptosis-related DEGs, “ConsensusClusterPlus” R package was used to perform unsupervised consensus clustering, an algorithm based on k-means machine learning. The consensus score and the relative change in area under the cumulative distribution function (CDF) curves confirmed the optimal classification number. Subsequently, Kaplan–Meier survival analysis was performed to assess the prognosis of patients with different clusters. In addition, the clinicopathological characteristics and the tumor immune microenvironment of PDAC patients in two clusters were further compared using “pheatmap” and “beeswarm” packages.

### Development and verification of prognostic risk signature

To evaluate the prognostic roles of PRGs, we further used univariate Cox regression analysis to identify the association between DEGs and survival status based on the TCGA cohort. The cut-off *P*-value was set as 0.15 to avoid omissions and then 12 survival-associated candidate genes were included in the least absolute shrinkage and selection operator (LASSO) Cox regression analysis (“glmnet” package) to narrow down and develop the prognostic risk signature. The prognostic signature was based on 10-fold cross-validation and the minimum criteria identified penalty parameter λ value. The risk score formula was calculated as follows: Risk score = $\sum_{i}^{n}{\mathrm{Coef}}_{i}*x_{i}$ (${\mathrm{Coef}}_{i}$: coefficients, $x_{i}$: gene-expression level). The PDAC patients were divided into either a high- or low-risk group according to the median risk score. The overall survival (OS) time of the two groups was calculated using Kaplan–Meier analysis and compared using log-rank test. The survival-dependent receiver-operating characteristic (ROC) curves at 1, 3, and 5 years were performed by employing the “survival,” “survminer,” and “timeROC” packages to determine the sensitivity and specificity of the prognostic risk signature. Principal component analysis was used for evaluating the ability of the risk signature to distinguish patients in different risk groups. The prognostic values of the risk signatures were also validated in the GSE62452 and GSE71729 merged database.

### Associations between pyroptosis-related genetic signature and clinical characteristics

The relationship between the risk scores and previously constructed molecular clusters was assessed using “pheatmap.” To explore the prognostic value of each signature gene, the optimal cut-off value was calculated by using the “survminer” package and Kaplan–Meier survival curves of OS for the nine hub genes were plotted.

The associations between individual genes’ signatures and patients’ clinical features (Supplementary Table 1) were conducted by using the “beeswarm” package. Univariate and multivariate Cox regression analyses were performed to evaluate the prognostic value of the pyroptosis-related genetic signature and selected clinical factors. Next, factors significantly associated with the prognosis of patients with PDAC in both univariate and multivariate analyses (*P *<* *0.05) were selected to plot a nomogram for the TCGA cohort using the “rms” R package. The calibration curves of the nomogram were illustrated to show the predicted OS of the nomogram against the observed rates.

### Functional enrichment and immune characteristics analyses

DEGs between the high- and low-risk groups were identified by using the “limma” package with a *P*-value* *<* *0.05. Gene Ontology (GO) and Kyoto Encyclopedia of Genes and Genomes (KEGG) analysis based on DEGs were performed by using the “clusterProfiler” package. Single-sample gene set enrichment analysis (ssGSEA) was conducted using the “gsva” package to calculate the scores of infiltrating immune cells. Also, the “estimate” package was employed to explore the differences in immune microenvironment between high- and low-risk groups. In addition, we calculated the tumor mutational burden (TMB) score for each patient with PDAC in the two groups. Furthermore, the expression status of common immune checkpoints was compared between high- and low-risk groups using boxplots.

### Statistical analysis

All statistical analyses were conducted by using R software (v4.1.0). Normally and non-normally distributed continuous data were compared by using the Student’s *t*-test and the Wilcoxon test, respectively. Chi-squared tests were performed for pairwise comparisons in categorical variables. The OS time between different groups was compared using Kaplan–Meier survival analysis with a log-rank test. The two-tailed *P*-value* *<* *0.05 was considered significant.

## Results

### Identification of DEGs between PDAC and normal pancreas tissues

**Figure 1.**
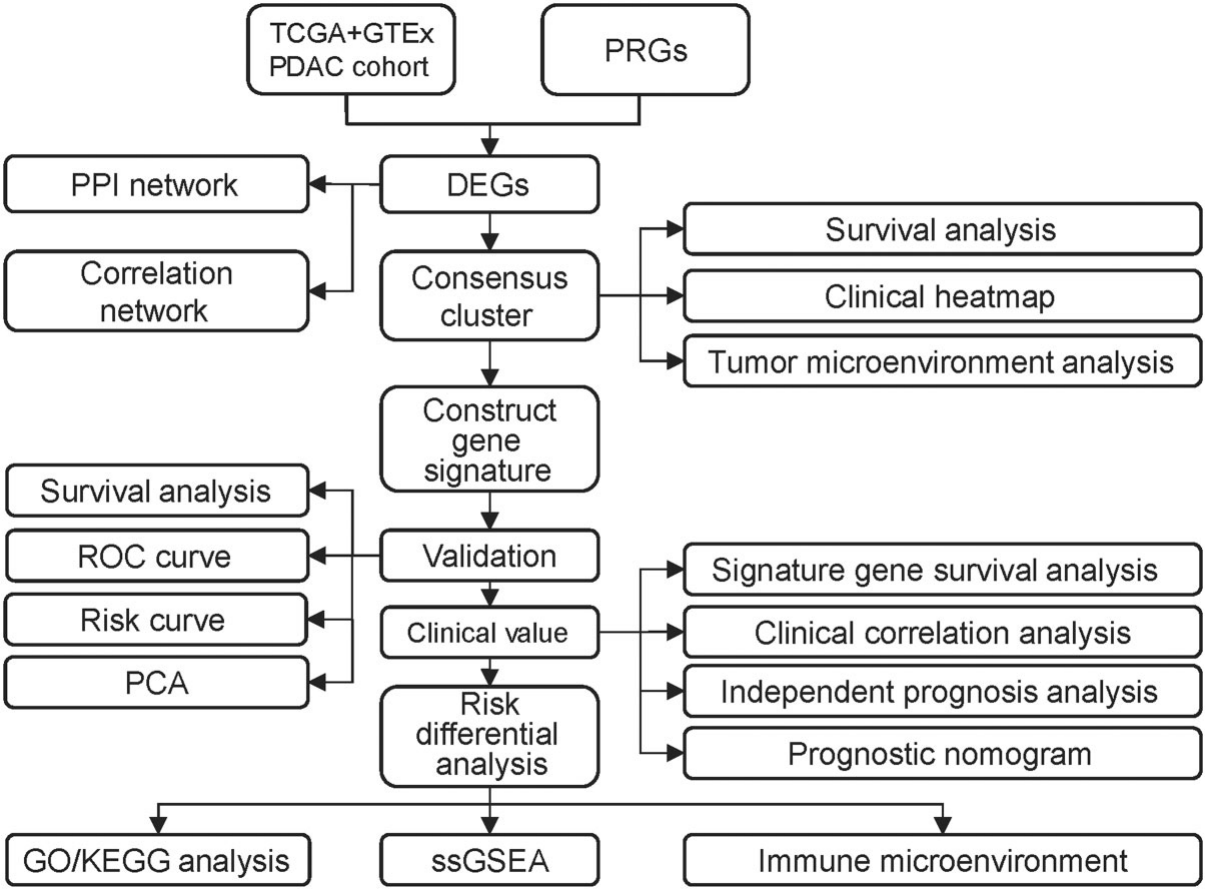
The flowchart of the study. PDAC, pancreatic ductal adenocarcinoma; TCGA, The Cancer Genome Atlas; GTEx, Genotype-Tissue Expression Project; PRGs, pyroptosis-related genes; DEGs, differentially expressed genes; PPI, protein–protein interaction; ROC, receiver-operating characteristic; PCA, principal component analysis; GO, Gene Ontology; KEGG, Kyoto Encyclopedia of Genes and Genomes; ssGSEA, single-sample gene set enrichment analysis.

The overview of the study flow chart is shown in Figure 1. A total of 171 normal and 179 tumor tissues with 57 pyroptosis-related gene expressions in TCGA and GTEx were included in this analysis. Among them, 29 DEGs, including 10 upregulated genes and 19 downregulated genes, were identified. The heat map illustrates the expression levels of these DEGs (Supplementary Figure 1A). To further explore the interactions between the 29 PRGs, we conducted a PPI network analysis (Supplementary Figure 1B). The correlation matrix diagram containing DEGs is presented in Supplementary Figure 1C.

### Tumor classification based on the PRGs

To explore the associations between the expression patterns of the 29 pyroptosis-related DEGs and PDAC subtypes, we constructed a consensus clustering analysis with all 176 PDAC patients in the TCGA cohort (Supplementary Figure 2A). According to the consensus CDF and relative change in area under the CDF curve, the optimal number of clusters was determined to be two (*k *=* *2). In this case, the intragroup correlations were strong, whereas intergroup correlations were weak; no apparent increase was found in the area under the CDF curve (Supplementary Figure 2B and C). Thus, the PDAC patients in the TCGA cohort could be well classified into two clusters (C1 and C2). The results of the Kaplan–Meier survival analysis indicated that patients in C2 exhibited significantly shorter survival time than patients in C1 (Supplementary Figure 2D).

**Figure 2.**
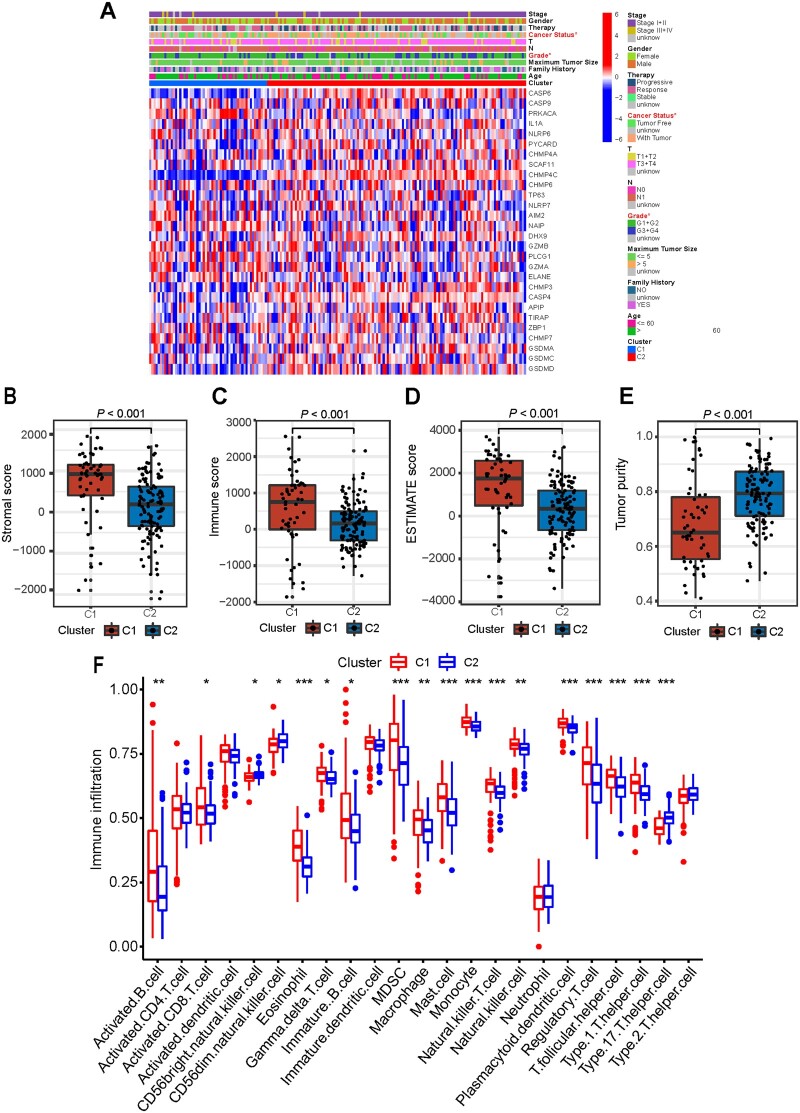
Characteristics of patients in different clusters. (**A**) Heat map and clinicopathological features C1 and C2 clusters and the significantly different clinical features are marked in red. (B)–(E) Comparison of stromal (B), immune (C), ESTIMATE (D) scores, and tumor purity (E) in C1 and C2 clusters. (F) The abundance of infiltrating immune cell types in C1 and C2 clusters.

The gene-expression profile and the clinical features between the two clusters are presented in a heat map (Figure 2A). We found that cancer status (with tumor or tumor-free) and pathological grade were significantly different between the two clusters. The differences in the immune microenvironment between the two clusters were further evaluated. The results revealed that the patients in C1 had significantly higher stromal score, immune score, and ESTIMATE score but lower tumor purity than those in C2 (Figure 2B–E). In addition, we analysed the abundance of 23 infiltrating immune cell types in the two clusters via the ssGSEA algorithm and found that innate immune cells including activated B cells, activated CD8^+^ T cells, eosinophils, γδ T cells, MDSCs, macrophages, mast cells, monocyte, natural killer cells, plasmacytoid dendritic cells, and T helper cells were remarkably richer in the patients in C1 than in those in C2 (Figure 2F).

### Development and verification of prognostic risk signature

To further explore the prognostic value of PRGs in the two clusters, we included 176 PDAC patients from TCGA with complete survival information for univariate Cox regression analysis to primarily screen the prognosis-related genes. The 12 genes (caspase-4 [*CASP4*], phospholipase C, gamma 1 [*PLCG1*], apaf-1 interacting protein [*APIP*], tumor protein p63 [*TP63*], charged multivesicular body protein 6 [*CHMP6*], gasdermin C [*GSDMC*], protein kinase cAMP-activated catalytic subunit alpha [*PRKACA*], charged multivesicular body protein 4C [*CHMP4C*], PYD and CARD domain containing [*PYCARD*], absent in melanoma 2 [*AIM2*], granzyme B [*GZMB*], and caspase-6 [*CASP6*]) that met the criteria of *P*-value* *<* *0.15 were chosen for further analysis (Supplementary Figure 3A). Next, we performed the LASSO Cox regression analysis with these 12 survival-associated candidate genes to identify the best-weighted coefficient genes for PDAC patients. The signature was determined according to the minimum criterion optimal λ value (Supplementary Figure 3B and C) and a group of nine genes were identified as the risk signature. The risk score was calculated as follows: risk score = 0.472 × expression level of *CASP4 *+* *(−0.569)* *× expression level of *PLCG1 *+* *(−0.321)* *×* *expression level of *APIP *+ (−0.256)* *×* *expression level of *CHMP6 *+* *0.355 × expression level of *GSDMC *+* *0.297 × expression level of *CHMP4C *+* *0.076 × expression level of *AIM2 *+* *0.092 × expression level of *GZMB *+* *0.121 × expression level of *CASP6*.

**Figure 3.**
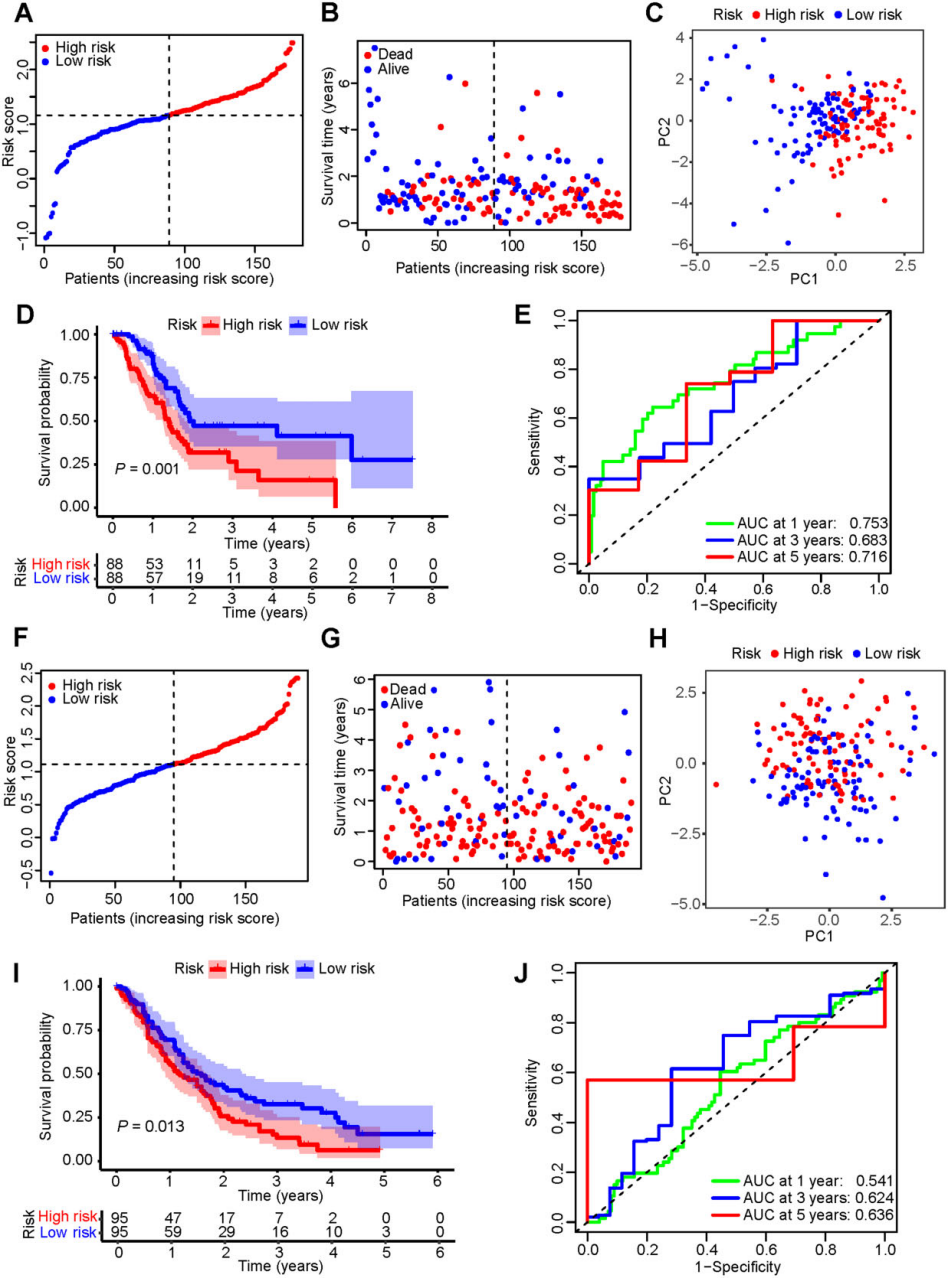
Prognostic performance of risk signature in the TCGA cohort and GEO validation set (GSE62452 and GSE71729). (A) and (F) The distribution of the patients based on the risk score. (B) and (G) The survival status analysis for each patient based on the risk score. (C) and (H) Principal component analysis (PCA) plot based on the risk score. (D) and (I) Kaplan–Meier survival curve of high- and low-risk groups. (E) and (J) Validation of time-dependent ROC curves at 1, 3, and 5 years of the prognostic value of the risk signature. TCGA, The Cancer Genome Atlas; GEO, Gene Expression Omnibus; ROC, receiver-operating characteristic; AUC, area under the curve.

Based on the median risk score calculated using the formula, all patients in the TCGA cohort were classified as in the high- or low-risk group (Figure 3A). Patients in the high-risk group had shorter survival times and more deaths than those in the low-risk group (Figure 3B). The patients in different risk groups could be well separated according to the principal component analysis (Figure 3C). Patients in the high-risk group had significantly shorter OS and higher mortality than those in the low-risk group (Figure 3D). Time-dependent ROC analysis was applied to evaluate the efficacy of the prognostic signature and we found that the area under the ROC curve (AUC) was 0.753 for 1-year, 0.683 for 3-year, and 0.716 for 5-year survival (Figure 3E). In the validation sets, similar results to those in the GSE62452 and GSE71729 merged database were observed (Figure 3F–J).

### Prognostic performance of nine signature genes in the TCGA cohort

**Figure 4.**
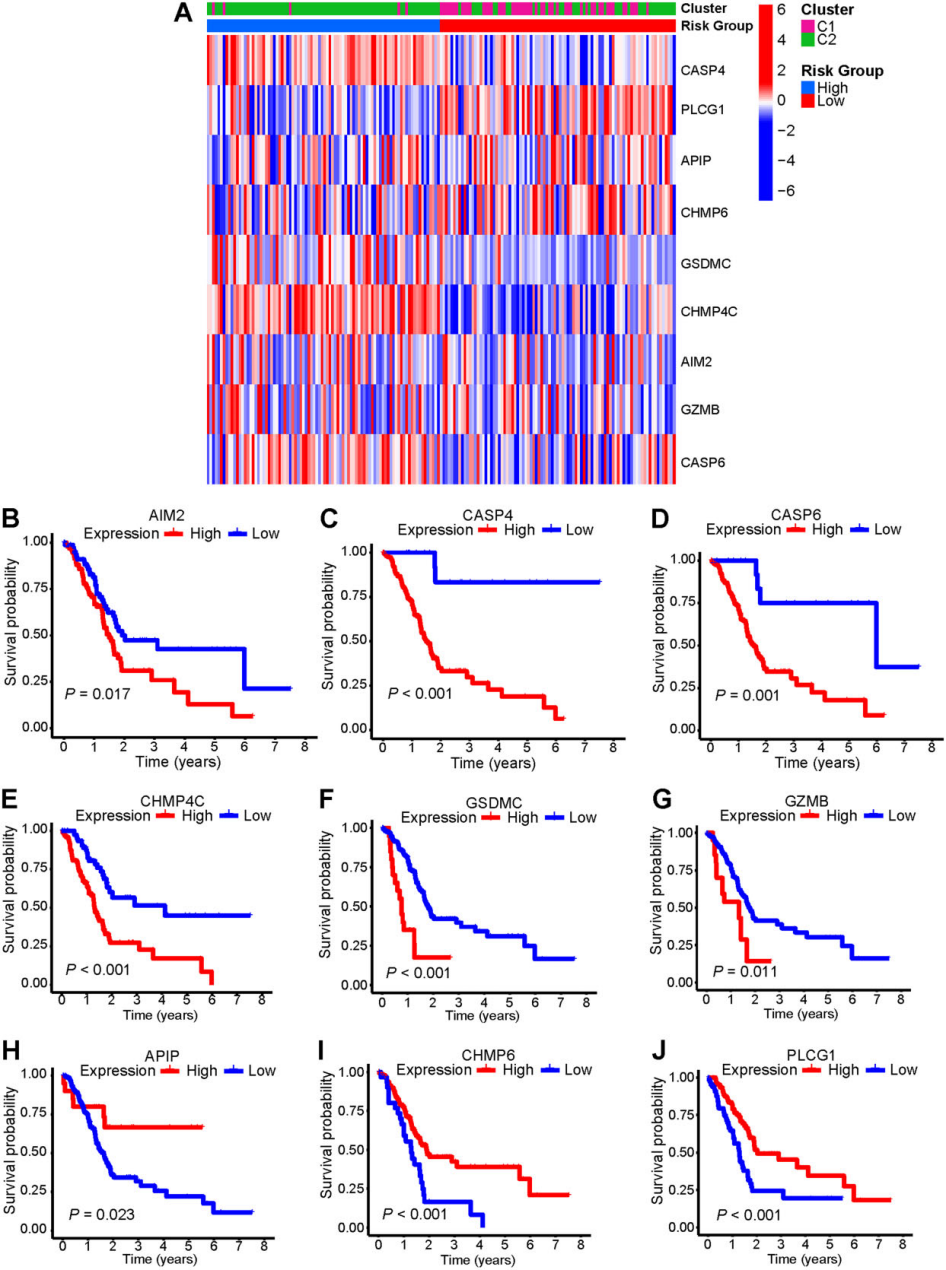
Prognostic performance of the signature genes in the TCGA cohort. (A) Heat map of pyroptosis-related signature genes by unsupervised clustering. The tumor classification and risk group as gene annotations are correlated. (B)–(J) Kaplan–Meier survival curve of each pyroptosis-related signature gene based on data from TCGA. TCGA, The Cancer Genome Atlas.

On the clustering heat map of the nine genes, the patients in C2 who had worse clinical outcome were mainly found in the high-risk group (Figure 4A), which was consistent with our finding shown above. We used the Kaplan–Meier survival curves to evaluate the prognostic values of the nine genes and the results showed that the expressions of *AIM2*, *CASP4*, *CASP6*, *CHMP4C*, *GSDMC*, and *GZMB* were negatively associated with survival, whereas the PDAC patients with lower expression of *APIP*, *CHMP6*, and *PLCG1* had poorer survival (Figure 4B–J).

### Association of risk model with clinical characteristics

**Figure 5.**
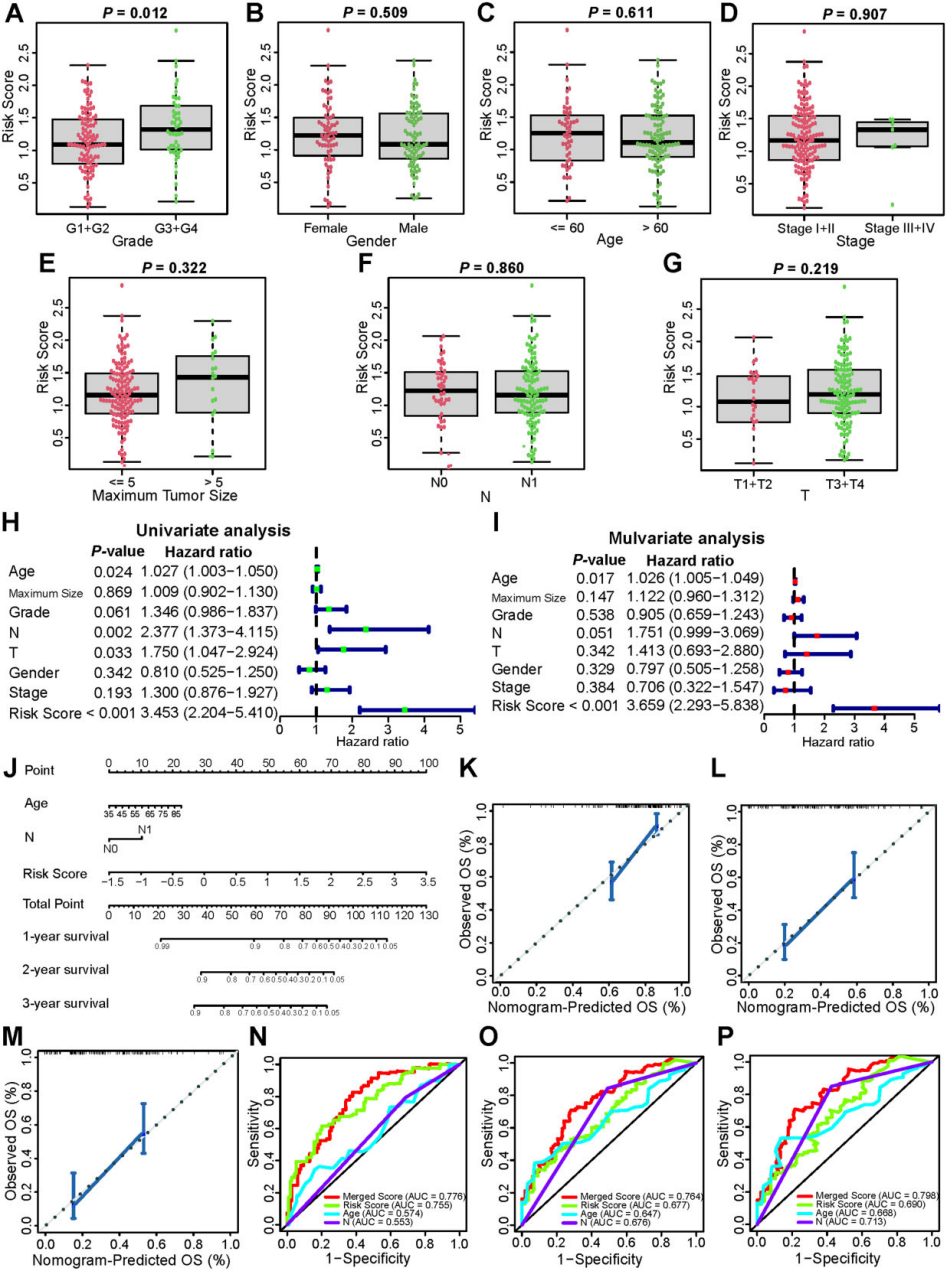
Association of risk models with clinical characteristics based on the TCGA cohort. The risk score was significantly associated with pathological grade (A) and was not significantly correlated with gender (B), age (C), stage (D), maximum tumor size (E), N stage (F), and T stage (G). (H) Univariate Cox regression analysis and (I) multivariate Cox regression analysis of clinical characteristics. (J) Nomogram to predict 1-, 2-, and 3-year OS of PDAC. (K)–(M) The calibration plot of the nomogram for internal validation. (N)–(P) The time-dependent ROC curves of the nomogram were compared based on 1- (N), 2- (O), and 3-year (P) OS in the TCGA cohort. TCGA, The Cancer Genome Atlas; AUC, area under the curve.

We performed the Wilcoxon rank-sum test to figure out the association between our risk model and the clinical characteristics of PDAC and found that the high-risk group had higher pathological grade diseases than the low-risk group (Figure 5A–G). To verify the accuracy of the risk model, we used univariate and multivariable Cox regression analyses to evaluate whether our risk model may work as a PDAC-independent prognostic factor (Figure 5H and I). The results indicated that the risk score was an independent predictor of poor survival (HR, 3.453; 95% CI, 2.204–5.410 and HR, 3.659; 95% CI, 2.293–5.838). Subsequently, we established a nomogram to predict patients’ OS with three independent prognostic factors (*P *<* *0.05): age, N stage, and risk score (Figure 5J). Calibration plots indicated that the nomogram was a well-fitted prediction model (Figure 5K–M). The AUCs of the nomogram were 0.776, 0.764, and 0.798 for 1-year, 2-year, and 3-year OS, respectively (Figure 5N–P).

### Functional enrichment analysis based on the risk model

**Figure 6.**
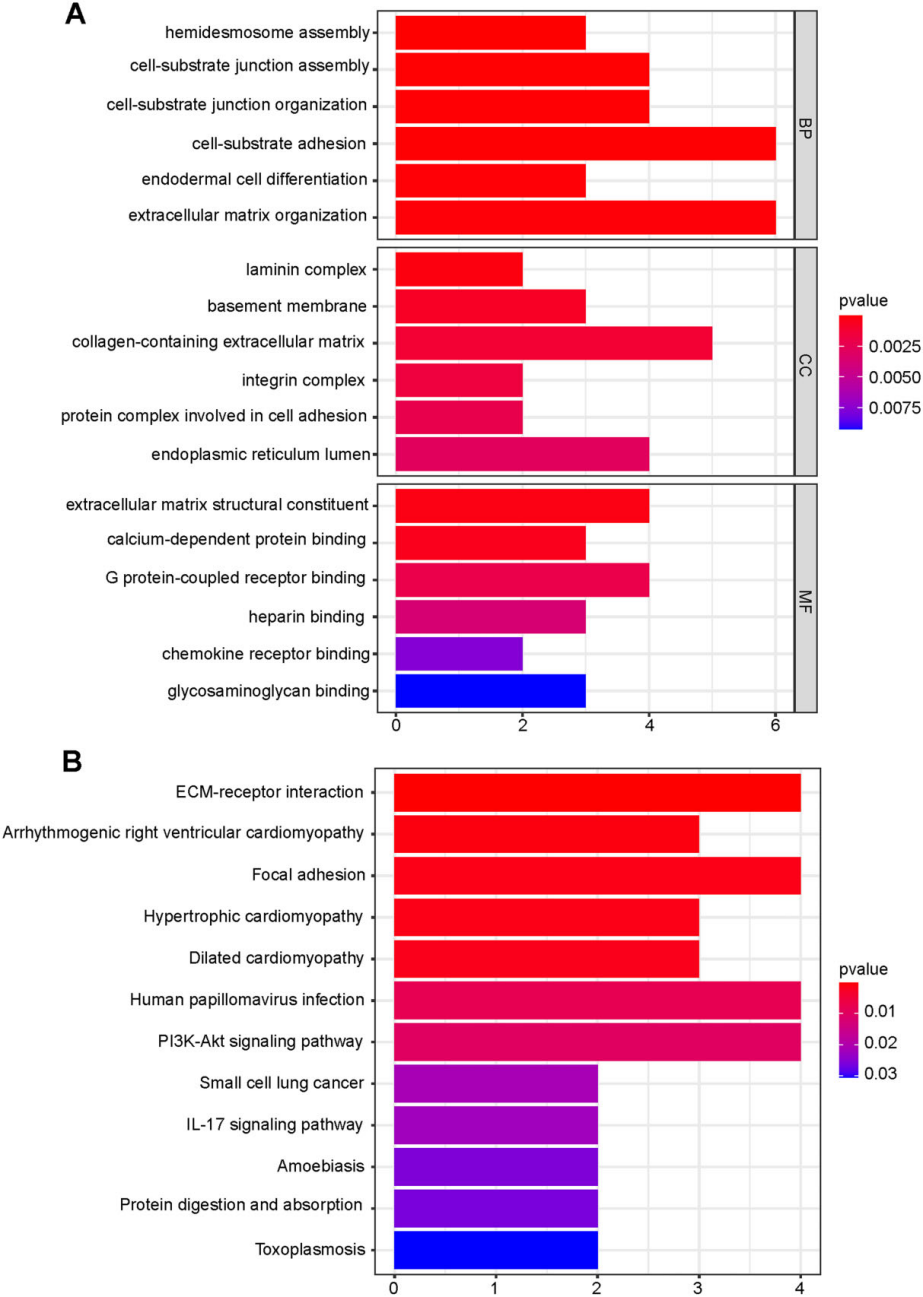
Functional enrichment analysis of differential genes between different risk groups. (A) Bar chart for GO enrichment. (B) Bar chart for KEGG pathways. GO, Gene Ontology; KEGG, Kyoto Encyclopedia of Genes and Genomes.

We performed the GO and KEGG analysis to further investigate the gene functions and pathways of the DEGs identified between the high- and low-risk groups. GO analysis suggested that these genes were mainly concentrated in cell-substrate adhesion, extracellular matrix organization, laminin complex, collagen-containing extracellular matrix, extracellular matrix structural constituent, chemokine receptor binding, etc. (Figure 6A). Also, KEGG analysis demonstrated that differential gene pathways were mainly involved the extracellular matrix (ECM)-receptor interaction, arrhythmogenic right ventricular cardiomyopathy, the PI3K-Akt signaling pathway, the IL-17 signaling pathway, etc. (Figure 6B).

### Relationship between risk score and immune infiltration

**Figure 7.**
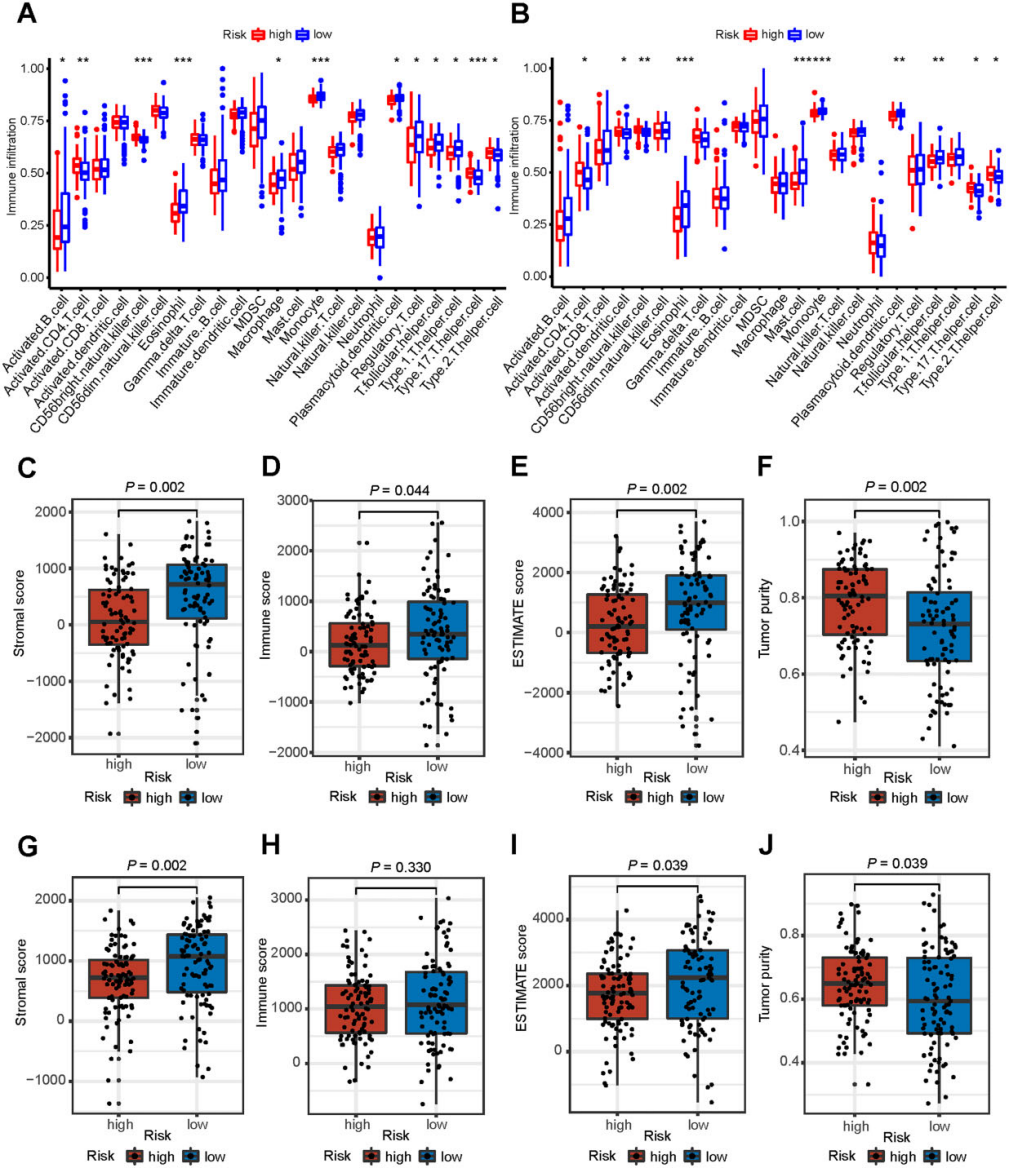
Relationship between risk score and immune infiltration in the TCGA cohort and GEO validation set (GSE62452 and GSE71729). (A) and (B) Comparison of the ssGSEA scores of 23 types of immune cells between high- and low-risk groups. (C)–(J) Comparison of stromal (C) and (G), immune (D) and (H), ESTIMATE (E) and (I) scores, and tumor purity (F) and (J) in the two groups. TCGA, The Cancer Genome Atlas; GEO, Gene Expression Omnibus.

**Figure 8.**
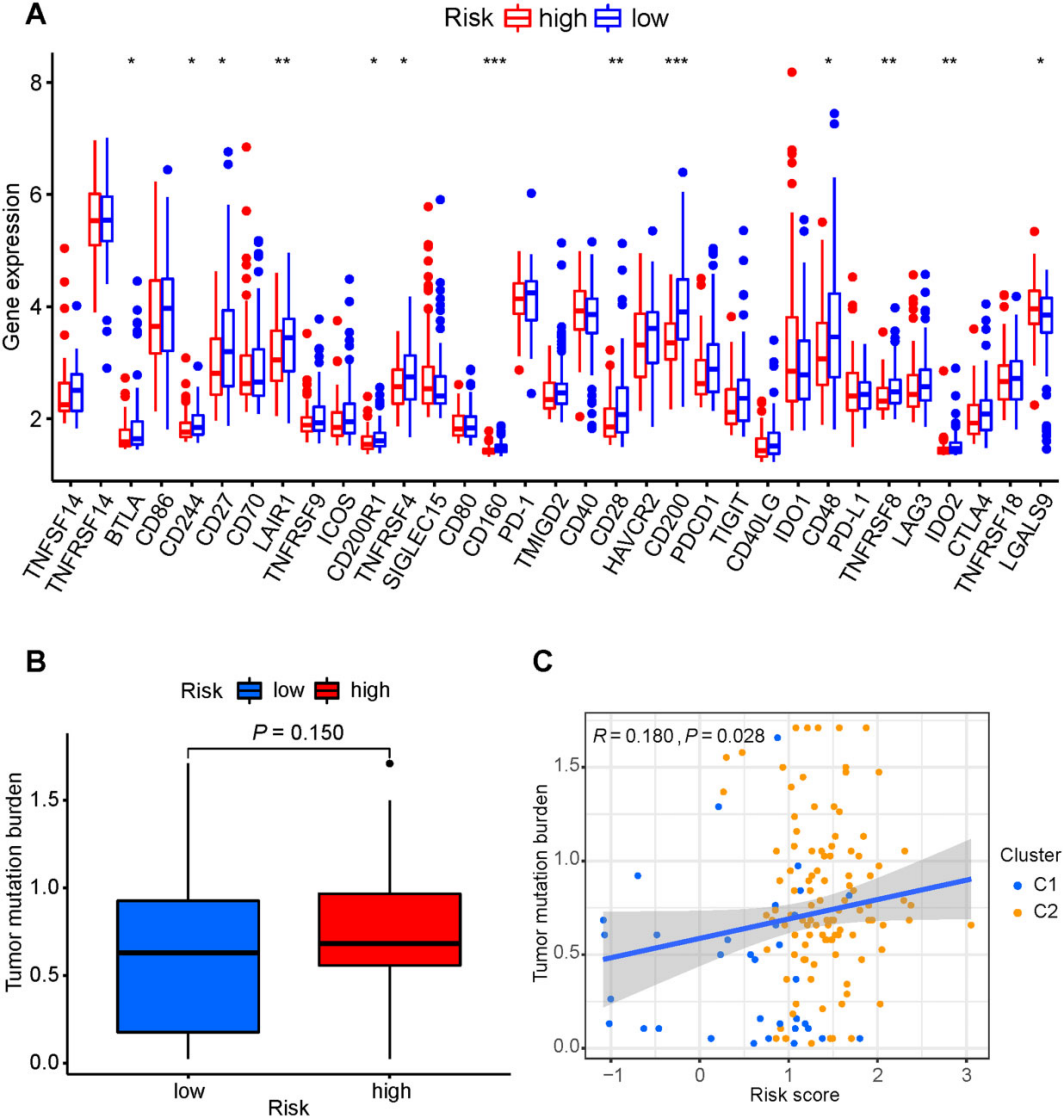
Evaluation of checkpoints and TMB between different risk groups. (A) Expression of immune checkpoints in the high- and low-risk groups. (B) TMB in high- and low-risk groups. (C) Spearman correlation analysis of the risk score and TMB. TMB, tumor mutational burden.

Our findings suggested that the risk scores may be useful in predicting clinical outcomes. We investigated whether risk score was indicative in treatment decisions, especially immunotherapy. We examined the 23 types of immune cells through the ssGSEA and the difference in the immune microenvironment between the two risk groups was further compared through the ESTIMATE algorithm. The results showed that the low-risk group generally had higher levels of infiltrating activated B cells, eosinophils, macrophages, monocytes, plasmacytoid dendritic cells, and type 1 T helper cells than the high-risk group (Figure 7A). The ESTIMATE algorithm showed that the stromal, immune, and ESTIMATE scores were higher in the low-risk group than in the high-risk group (Figure 7C–E), whereas tumor purity was higher in the high-risk group than in the low-risk group (Figure 7F). Validation was performed using the GSE62452 and GSE71729 databases, and similar results were observed (Figure 7B and G–J). The expressions of the genes in immune checkpoints including *BTLA*, *CD160*, *CD200*, and *IDO2* in the low-risk group were notably higher than those in the high-risk group. However, no statistical difference was observed in *PD-1/PD-L1* between the two groups (Figure 8A). Given the clinical importance of the TMB, we analysed the inherent relevance between the TMB and risk score. Although statistically insignificant, the high-risk group patients tended to have a greater TMB than the low-risk group (Figure 8B). Correlation analysis confirmed that the risk score was positively correlated with the TMB (Spearman coefficient: *r *=* *0.18, *P *=* *0.028; Figure 8C). Overall, our results showed that the PDAC patients in the low-risk group had higher activity in the immune microenvironment and lower TMB than those in the high-risk group.

## DISCUSSION

Pyroptosis, an inflammatory and programmed cell death, has been found to play an essential role in the development of various malignancies. It appeared to be significantly related to tumor proliferation, angiogenesis, migration, invasion, and recurrence via regulating a series of essential signal pathways to influence the patients’ prognosis [15]. However, the prognostic values and the involved mechanisms of PRGs in PDAC have not been fully elucidated.

In this study, one nine-gene risk signature was generated via LASSO Cox regression analysis, which was validated in the external data set (GSE62452 and GSE71729 merged database). A nomogram was then established to predict the 1-, 2-, and 3-year OS of PDAC patients. The functional enrichment analyses revealed that pyroptosis might regulate the tumor immune microenvironment of PDAC. Immune infiltration evaluation suggested that immune status was more activated in the low-risk group than in the high-risk group. The TMB was also significantly positively correlated with risk scores. Our study may provide new evidence for prognostic biomarkers and therapeutic targets for PDAC.

When analysing the tumor immune microenvironment in the two tumor clusters, we found that the C1 cluster had a higher immune score and lower tumor purity with better survival. Besides, the antitumor immune cells, such as CD8^+^ T cells, activated B cells, and natural killer T cells, were upregulated in the C1 cluster. The reason might be that lower tumor purity, consistent with a higher immune score, was related to more robust immune responses, leading to a longer OS [16].

By LASSO analysis, we identified nine signature genes (*APIP*, *CHMP6*, *PLCG1*, *CASP4*, *CASP6*, *CHMP4C*, *GSDMC*, *AIM2*, and *GZMB*). *APIP* has been proven to inhibit two types of programmed cell death, including apoptosis and pyroptosis, and was also found to be related to cancers and inflammatory diseases [17]. The previous study demonstrated that the expression of uveal autoantigen with coiled-coil domains and ankyrin repeats (*UACA*) and *APIP* was downregulated in non-small cell lung cancer, leading to the loss of Apaf-1 nuclear entry assisted by *UACA*, which may underlie the failure of DNA-damage checkpoint activation, causally contributing to the development and progression of small cell lung carcinoma [18]. This might explain the fact that the PDAC patients with lower expression of *APIP* in the TCGA cohort had poorer survival. *CHMP6* encodes a member of the chromatin-modifying protein/charged multivesicular body protein family. Overexpression of *CHMP6* would cause cell death, mainly via oncosis and to a certain extent via apoptosis in HeLa cells [19]. *PLCG1* was found to contribute to gasdermin D-mediated pyroptosis through a calcium-dependent mechanism [20]. Recently, Saliakoura *et al*. [21] found that *PLCG1* suppression in hypoxia resisted apoptosis, elevated lung adenocarcinoma cancer cell proliferation, and predicted poor patient survival. The expressions of the other six genes (*CASP4*, *CASP6*, *CHMP4C*, *GSDMC*, *AIM2*, and *GZMB*) in PDAC patients in our study were negatively associated with survival. *CASP4* plays a vital role in pyroptosis as the primary mediator of non-canonical pathways [22]. Interestingly, evidence indicated that elevated expression of *CASP4* was associated with poor survival in patients with renal cancer [23]. *CASP6* plays an essential role in promoting the activation of programmed cell death, including pyroptosis and apoptosis [24]. However, few studies have focused on *CASP6* in PDAC and the mechanism needs further investigation. Upregulation of *CHMP4C* has been reported in cervical cancer and it is associated with poor survival [25]. *GSDMC* is a member of the GSDM family. Hou *et al*. [26] found that p-Stat3 interacted with *PD-L1* and facilitated its nuclear translocation, which in turn promoted *GSCMC*/*CASP8* activation and mediated the non-canonical pyroptosis. Moreover, overexpression of *GSDMC* is associated with poorer survival in breast cancer [26], which is consistent with our findings. *AIM2* can promote the release of IL-1β and IL-18, thereby promoting pyroptosis [27]. The role of *AIM2* may be controversial in different cancer types; in our study, upregulation of *AIM2* was associated with shorter OS time in PDAC, consistently with that in nasopharyngeal carcinoma and non-small cell lung cancer [28]. *GZMB*, a component of our risk signature, is involved in the cleavage of gasdermin E that directly leads to pyroptosis [29]. Similarly to the finding in cervical cancer [30], *GZMB* expression was negatively associated with survival in patients with PDAC in the present study.

To further explore the mechanisms of the risk signature, we revealed that DEGs between the high- and low-risk groups are mainly involved in immune-response pathways, indicating that pyroptosis might regulate the tumor immune microenvironment of PDAC. Hence, we next analysed the immune status in different risk groups. We noticed that the immune microenvironment characteristics of different risk groups were highly consistent with those of patients in different clusters mentioned above—that is, patients in the high-risk group with poorer prognoses had lower immune scores and higher tumor purity in the immune microenvironment than those in the low-risk group. It can be explained easily by the consensus that PDAC is an immunosuppressive tumor [31]. When considering the ssGSEA analysis, we also found that the number of most antitumor immune cells declined in the high-risk group, suggesting immune-function deficiency and poor survival, which was consistent with previous studies in PDAC [32].

Immune checkpoint blockade (ICB) therapy targeting *CTLA-4* or *PD-1/PD-L1* has a limited effect in advanced PDAC. The lacking of strong pre-existing T-cell immunity may underlie the failure of ICB therapy for PDAC [33]. No significant difference was observed in *PD-1/PD-L1* expression between the different risk groups in our study. However, the association between *PD-L1* expression and response to ICB therapy in PDAC is still unclear. The patients in the low-risk group with more antitumor immune cells might benefit from a combinatorial approach of immunotherapy with other modalities. Although TMB is considered an effective biomarker for predicting immunotherapy response in patients with solid tumor, its effectiveness in some immunologically cold tumors, such as PDAC, remains controversial. Our results showed that patients in the low-risk group with lower TMB had better survival than those in the high-risk group with higher TMB. This may be due to the negative correlation between TMB and many anticancer signatures, such as CD8^+^ T cells, macrophages, and T helper cells [34].

The present study has several limitations. First, a series of newly identified PRGs need to be incorporated to optimize the accuracy of the present model. Second, the present study was conducted on data acquired from public databases so the results might be influenced by an inherent case-selection bias, and further studies with *in vitro* and *in vivo* research are needed to confirm our findings. Moreover, due to the limited resources of detailed clinical features available from public databases, treatment methods were not included in the OS analysis and the nomogram could not be verified in the GEO database. In addition, currently, there is a lack of sufficient data from patients without surgery in the TCGA and GEO databases, so we were unable to assess the nine-gene scoring system in non-resected PDACs.

## Conclusions

In summary, our study provided a good prognostic risk signature consisting of nine PRGs for patients with PDAC. Notably, we constructed a prognostic nomogram to assist clinical treatment decisions. Immune characteristics analysis between different risk groups demonstrated that pyroptosis might affect the prognosis of patients with PDAC via regulating the tumor immune microenvironment. Our findings suggest that pyroptosis might be a promising therapeutic target for PDAC.

## Supplementary Data

Supplementary data is available at *Gastroenterology Report* online.

## Authors’ Contributions

X.Y.Y. conceived and designed the project. X.X. collected the data. X.X., J.H.L., J.H.L., and Q.C.X. analysed and interpreted the data. X.X., J.H.L., J.H.L., and Q.C.X. drafted the manuscript. X.Y.Y. revised the manuscript as the corresponding author and provided comments. All authors read and approved the final manuscript.

## Funding

This study was supported by the National Natural Science Foundation of China [grant no. 82072644 and no. 81772522].

## Supplementary Material

goac051_Supplementary_Data
