# Supplementary material for: Values of a novel pyroptosis-related genetic signature in predicting outcome and immune status of pancreatic ductal adenocarcinoma
Source: Gastroenterol Rep (Oxf). 2022 Sep 29;10:goac051. doi: 10.1093/gastro/goac051 (PMC9522386; doi:10.1093/gastro/goac051)
Supplement: goac051_Supplementary_Data [file goac051_supplementary_data.pdf]

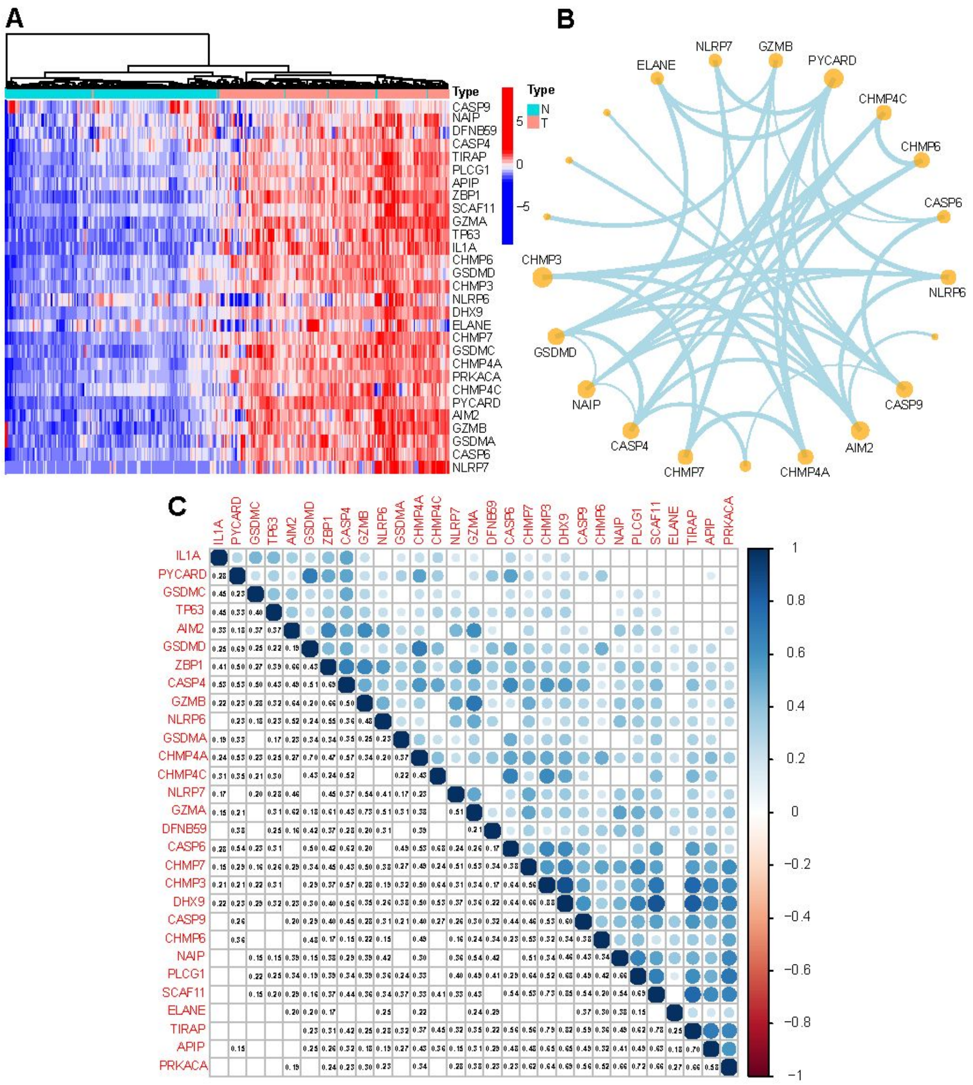

Supplementary Figure 1. Expressions of the pyroptosis-related genes and the interactions between them.

(**A**) Heatmap of differentially expressed PRGs between PDAC and normal pancreas tissues. (**B**) PPI network to explore the interactions of the PRGs (The size of nodes represents the number of associated genes, and the width of the line is related to the score of protein interaction. Only show gene names with more than 3 connected nodes). (**C**) The correlation matrix diagram of the pyroptosis-related DEGs. Nonsignificant correlations (*P* value > 0.05) are not shown in this figure. PRGs, pyroptosis-related genes; PPI, protein-protein interaction; DEGs, differentially expressed genes.

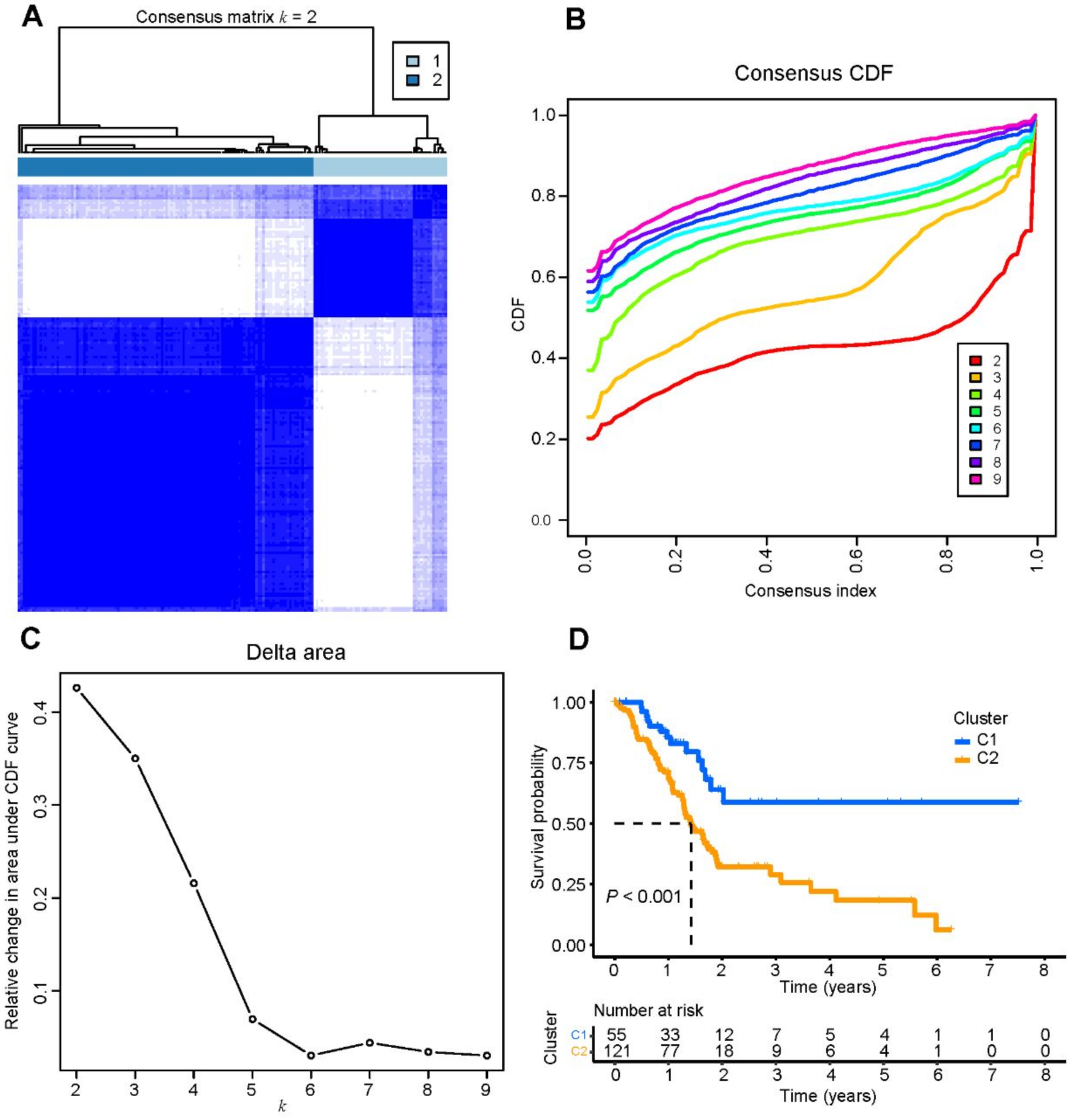

Supplementary Figure 2. Tumor classification based on the differentially expressed PRGs.

(**A**) Patients are divided into two clusters according to the consensus matrix (*k* = 2). (**B**) CDF curves of the consensus value (*k*, 2 to 9). (**C**) Relative change in area under the CDF curve when cluster number changes from *k* to *k*+1. The range of *k* is from 2 to 9. (**D**) Kaplan-Meier survival analysis of the patients in two clusters. PRGs, pyroptosis-related genes; CDF, cumulative distribution function.

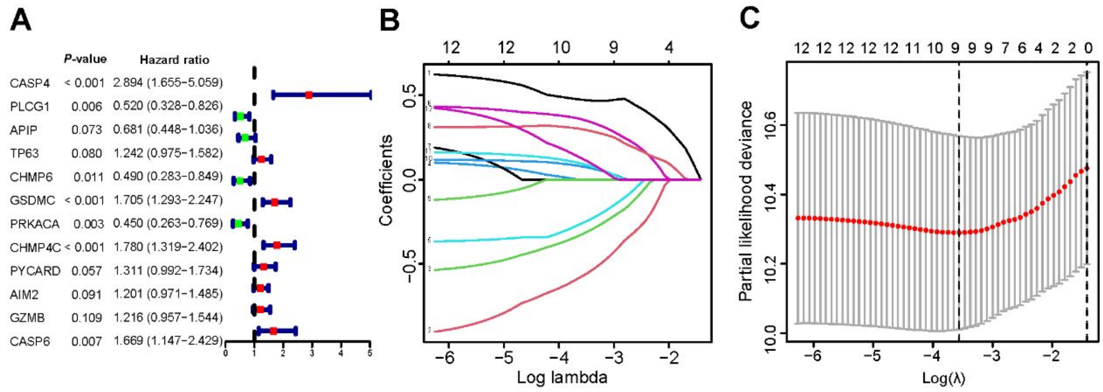

Supplementary Figure 3. Development of risk signature in the TCGA cohort.

(**A**) Univariate Cox regression analysis of OS for DEGs. (**B**) LASSO regression of the 12 OS-related genes. (**C**) Cross-validation for tuning the parameter selection in the LASSO regression. TCGA, The Cancer Genome Atlas; OS, overall survival; DEGs, differentially expressed genes; LASSO, least absolute shrinkage and selection operator.

Supplementary Table 1. The clinicopathological characteristics of 176 PDAC patients in the TCGA cohort.

Characteristic	Value
Age, years (mean ± SD)	64.7±10.8 (35-88)
Race (Asian/African American/White/Not reported)	11/6/155/4
Sex (Female/Male)	80/96
Maximum tumor size, cm (≤ 5/ > 5/Not reported)	142/21/13
T stage (T1/T2/T3/T4/TX)	7/24/140/3/2
N stage (N0/N1/NX)	49/122/5
M stage (M0/M1/MX)	79/4/93
TNM stage (I/Ⅱ/Ⅲ/Ⅳ/Not reported)	21/145/3/4/3
Histologic grade (G1/G2/G3/G4/Gx)	30/94/48/2/2
Surgery type (Distal pancreatectomy/Total pancreatectomy/Whipple/Other methods)	23/3/135/15
Radiation therapy (Yes/No/Not reported)	37/100/39
Family history of cancer (Yes/No/Not reported)	62/47/67
Cancer status (Tumor free/With tumor/Not reported)	44/82/50
History of chronic pancreatitis (Yes/No/Not reported)	13/127/36
History of diabetes (Yes/No/Not reported)	38/107/31
